# Parasites rather than phoronts: *Teratorhabditis synpapillata* nematodes reduce lifespan of their *Rhynchophorus ferrugineus* host in a life stage‐dependent manner

**DOI:** 10.1002/ece3.8004

**Published:** 2021-08-09

**Authors:** Manel Ibrahim, Ameni Loulou, Anissa Brouk, Arthur Muller, Ricardo A. R. Machado, Sadreddine Kallel

**Affiliations:** ^1^ Laboratoire de Recherche Bio‐agresseur et Protection Intégrée en Agriculture, LR14GR02 Université de Carthage National Agronomic Institute of Tunisia Ariana Tunisia; ^2^ Experimental Biology Research Group Faculty of Sciences Institute of Biology University of Neuchâtel (UniNE) Neuchâtel Switzerland

**Keywords:** ecological interactions, insect pests, insect–nematode interaction, parasitic nematodes, phoresy, red palm weevils

## Abstract

*Rhynchophorus ferrugineus* Olivier (Coleoptera: Curculionidae) red palm weevils are often reported in association with different organisms including nematodes. The significance of this interaction and whether nematodes can influence their life‐history traits is unclear. We collected *Rhynchophorus ferrugineus* red palm weevils at different developmental stages and locations in Tunisia, observed and dissected them in search for nematodes and other interacting organisms, established laboratory colonies and identified the nematodes associated with them, and conducted nematode–insect interaction assays to determine the capacity of the nematodes to influence their life‐history traits. We observed *Beauveria bassiana* fungi in larvae, nymph, and adults; *Centrouropoda* and *Uroobovella* acari associated with the adults, and *Teratorhabditis synpapillata* nematodes associated with larvae and adults. Nematode–insect interaction bioassays revealed that *T*. *synpapillata* nematodes reduce the lifespan of the insect larvae in a population‐dependent manner, but do not influence the lifespan of adults. Our study uncovers an important factor that may determine population dynamics of this important palm pests and provides evidence to conclude that these organisms establish a parasitic relationship, rather than a phoretic relationship.

## Graphical abstract

Palm Red Weevil Interact with nematodes and associated mites. *Teratorhabditis synpapillata* nematodes reduce lifespan of their *Rhynchophorus ferrugineus* host in a life stage‐dependent manner.

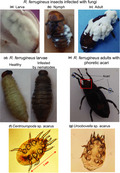

## 1 INTRODUCTION

The red palm weevil, *Rhynchophorus ferrugineus* (RPW) (Olivier, 1790) Herbst, 1795 (Coleoptera, Curculionidae), is one of the most voracious pest of palm trees, including *Phoenix canariensis* Hort. ex Chabaud (Liliopsida: Phoeniceae) date palms, worldwide. It is originally from southern India, but it has currently invaded Mediterranean countries including Tunisia, where it was reported in 2011 (Chebbi, 2011; Cox, 1993; Rochat et al., 2017).

*Rhynchophorus ferrugineus* weevils are often reported in association with many different organisms, including acari and nematodes (Abolafia & Ruiz‐Cuenca, 2020; Kanzaki et al., 2008; Peter, 1989). In Tunisia in particular, red palm weevils interact with phoretic uropodid mites. Three species have been reported: *Uroobovella javae* attached to the antenna, thorax, and legs; *Centrouropoda almerodai* found beneath elytra; and *Uroobovella marginata* attached to the pygidium, thorax, and head of red palm weevil adults (Slimane‐Kharrat & Ouali, 2019). Many other acari species have also been reported in other countries including *Trichouropoda* sp., *Macrocheles mammifer*, *U. assamomarginata*, and *U. javae* (Abolafia & Ruiz‐Cuenca, 2020; Dilipkumar et al., 2015; Hassan et al., 2011; Porcelli et al., 2009). Similarly, several species of nematodes establish different types of associations with red palm weevil insects including species of the genus *Acrostichus*, *Bursaphelenchus*, *Caenorhabditis*, *Diplogasteritus*, *Mononchoides*, and *Teratorhabditis* (Camerota et al., 2016; Troccoli et al., 2015).

In contrast with acari that are broadly accepted to establish phoretic association with red palm weevil insects, the type of association established by the nematodes and these insects is less clear. In the case of *Teratorhabditis synpapillata* nematodes in particular, they were initially considered a bacterial‐feeding species (Kanzaki et al., 2008; Sudhaus, 1985; Tahseen & Shamim Jairajpuri, 1988; Yeates et al., 1993). Later, this nematode species was isolated from *Rhynchophorus ferrugineus* red palm weevils and classified as a phoretic nematode, as they were isolated under elytra of adults insect in Japan (Kanzaki et al., 2008). Phoresy is a type of commensalism in which an organism of smaller size attaches itself to another one with the only purpose of increasing its dispersal potential (White et al., 2017). Thus, a phoretic commensal interaction strictly implies no cost for the host and a fitness benefit for the phoront. As insects serve as transporters and assuming that they do not incur in any fitness consequences by providing this service, and nematodes benefit from being dispersed into new environments, this type of nematode–weevil interaction is often classified as phoretic commensalism. However, there is a number of considerations that speak against this notion. First, depending on the densities, nematodes attached to the adult insects can impair flight behavior, thus, conveying costs. Secondly, when transported into the nest, nematodes can reduce insect’ available resources as nematodes and insects feed from the same food: rooted plant tissues. Third, nematodes can invade different insect’ internal organs, and even reach the hemocoel, impairing thereby insect physiological processes, that could impact their lifespan. Thus, experimental evidence for the type of relationship established between these organisms is required if we are to understand the ecology and evolution of this insect–nematode interaction.

In this study, we collected *Rhynchophorus ferrugineus* red palm weevils at different developmental stages in different locations of Tunisia. We observed, inspected, and dissected the insects to characterize the organisms that naturally interact with them. In addition, we conducted laboratory experiments to gain insights into the type of relationship that they establish, particularly the relationship established between nematodes and larvae and adult weevils. We found that the nematodes reduce the lifespan of red palm weevils in a life stage‐dependent manner, uncovering the parasitic lifestyle of *T. synpapillata* nematodes. Our study reveals an important factor that may determine population dynamics of this important pests in Tunisia.

## 2 MATERIAL AND METHODS

### 2.1 Insect collection

**FIGURE 1:**
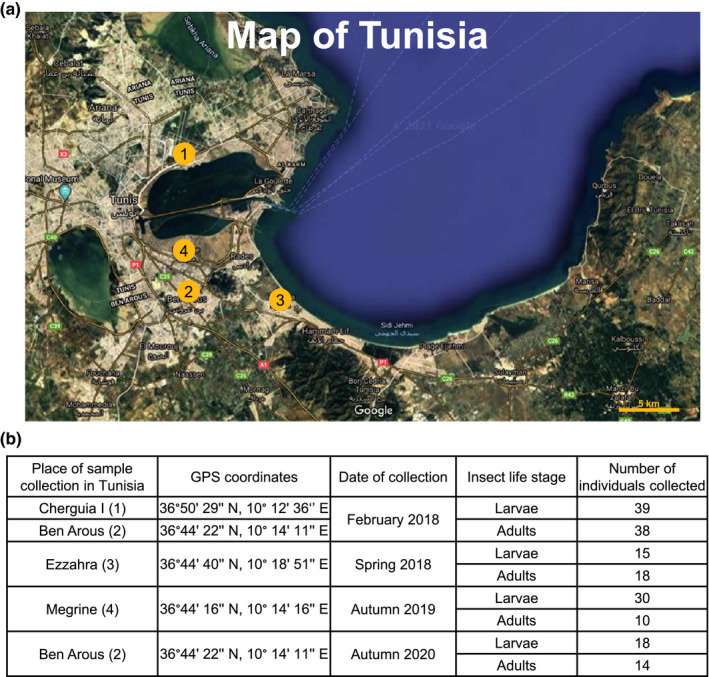
Geographical location of insect sampling sites. (a) Insect samples were collected from *Phoenix canariensis* palm trees at four different locations in Tunisia: Cherguia I (1), Ben Arous (2), Ezzahra (3), and Megrine (4). (b) Place of sample collection, GPS coordinates, date of sample collection, insect life stage collected, and number of individuals collected

To characterize the organisms that naturally interact with *Rhynchophorus ferrugineus* red palm weevils, we carried out four insect collection campaigns and collected *Rhynchophorus ferrugineus* insects at the larval stage (4th‐5th instar, as determined by the width of the head capsule, 5 mm in average, according to Dembilio & Jacas, 2011) and at the adult stage from *Phoenix canariensis* ornamental palm trees in different locations of Tunisia (Figure 1a). During the first one, in February 2018, 38 adults and 39 larvae were collected in the cities of Charguia I (Tunis, Tunisia) and Ben Arous (Ben Arous, Tunisia). During the second one, in spring 2018, 15 adults and 18 larvae weevils were collected from Ezzahra (Ben Arous, Tunisia). During the third campaign, in autumn 2019, 10 adults and 30 larvae were collected from Megrine (Ben Arous, Tunisia). The last collection campaign, carried in autumn 2020 in Ben Arous (Ben Arous, Tunisia), yielded 14 adults and 18 larvae. All insects were placed individually in Petri plates, transferred to the laboratory, and studied as described below.

### 2.2 Characterization of nematodes, fungi, and acari naturally associated with red palm weevils

To determine the organisms that naturally interact with red palm weevils, we inspected living insects in search for acari and dissected larval and adult cadavers in search for fungi and nematodes. To this end, all the insects collected in spring 2018 in the cities of Cherguia I, Ben Arous, and Ezzahra were transferred individually to Petri plates (90 mm diameter) layered with humidified filter papers and maintained at 25℃. During the incubation period, some insects showed strong symptoms of fungi infection and died. To identify the potential causative agent of death, infected larvae and adults were transferred to PDA media and incubated at 25℃ for 1 week. Mycelia and conidia were stained with methylene blue and observed under a light microscope (Olympus, AX9602) for identification. To isolate and identify the acari associated with red palm weevil adults, adult elytra were carefully inspected and acari were captured and conserved in alcohol 50%. After this, the acari were cleared by incubating them in lactic acid at ambient temperature for 1 week. Then, acari were transferred to Hoyer media and observed under a light microscope (Olympus, AX9602). To isolate the nematodes associated with red palm weevils, nematodes present in the Petri plates were harvested and disinfected twice with a solution of 7‰ of streptomycin dihydrosulfate and a solution of 1‰ of mercuric chloride (HgCl_2_) followed by three immersions in sterile water using a fine mesh sieve (5 μm). Two populations of nematodes were obtained CrpL and CrpA from larvae and adults of RPW, respectively. Laboratory colonies of these two nematode isolates were established by culturing the nematodes in egg yolk medium (32 g egg yolk and 24 g agar per liter distilled water).

### 2.3 Nematode identification

To determine the taxonomic status of the two nematode populations, CrpL and CrpA, isolated from the larvae and the adults of red palm weevils, molecular identification based on rRNA gene sequences was carried out (Bruno et al., 2020; Fallet et al., 2020). For this, nematode genomic DNA was extracted using the genomic DNA isolation kit from Norgen Biotek (Thorold, Ontario, Canada) following the manufacturer's instructions. Genomic DNA isolated was used to PCR amplify different regions of the rRNA genes. ITS regions (ITS1, 5.8S, ITS2) were amplified using primers 18S: 5′‐TTGATTACGTCCCTGCCCTTT‐3′ (forward), and 26S: 5′‐TTTCACTCGCCGTTACTAAGG‐3′ (reverse) (Vrain et al., 1992). The fragment containing the D2/D3 regions of the 28S rRNA gene was amplified using primers D2F: 5′‐CCTTAGTAACGGCGAGTGAAA‐3′ (forward) and 536:5′‐CAGCTATCCTGAGGAAAC‐3′ (reverse) (Nadler et al., 2006). The 18S rRNA gene was amplified using primers NEM18SF: 5′‐CGCGAATRGCTCATTACAACAGC‐3′ (forward) and NEM18SR: 5′‐GGGCGGTATCTGATCGCC‐3′ (reverse) (Floyd et al., 2005). PCR cycling conditions used were as follows: an initial denaturation step at 98℃ for 10 min, annealing at 58℃ for 30 s, and extension at 72℃ for 90 s. PCR products were separated by electrophoresis (45 min, 100 V) in a 1% TAE (Tris–acetic acid–EDTA) buffered agarose gel stained with GelRed nucleic acid gel stain (Biotium). PCR products were sent to Microsynth AG (Balgach, Switzerland) for Sanger sequencing. Sequences were manually curated and trimmed. All sequences were deposited in the National Centre for Biotechnology Information (NCBI) databank. Accession numbers are given in the phylogenetic trees and summarized in Table S1.

### 2.4 Nematode phylogenetic relationships reconstruction

The evolutionary histories based on the different rRNA gene sequences were inferred by using the maximum likelihood method based on the general time reversible model (18S and ITS) or on the Kimura 2‐parameter model (D2D3) (Kimura, 1980; Nei & Kumar, 2000). Best‐fit substitution model analyses were carried out prior to inferring evolutionary histories (Kumar et al., 2016; Nei & Kumar, 2000). In all cases, the trees with the highest log‐likelihood are shown. The percentage of trees in which the associated taxa clustered together is shown next to the branches. Initial tree(s) for the heuristic search were obtained automatically by applying neighbor‐joining and BioNJ algorithms to a matrix of pairwise distances estimated using the maximum composite likelihood (MCL) approach and then selecting the topology with superior log‐likelihood value. A discrete Gamma distribution was used to model evolutionary rate differences among sites (5 categories). The trees are drawn to scale, with branch lengths measured in the number of substitutions per site. Evolutionary analyses were conducted in MEGA7 (Kumar et al., 2016). Graphical representation and editing of the phylogenetic trees were performed with the Interactive Tree of Life (version 3.5.1) (Chevenet et al., 2006; Letunic & Bork, 2016).

### 2.5 Nematode–insect interaction assays

To gain insights into the type of symbiosis between the larvae and adults of the red palm weevils *Rhynchophorus ferrugineus* and the nematodes isolated from them and to evaluate whether these nematodes alter the lifespan of the insects, we observed the interaction between these organisms for several days and recorded insect mortality overtime. For this, we used the larvae and adults of the red palm adult weevils collected in Megrine (Autumn 2019, 30 larvae and 10 adults) and Ben Arous (Autumn 2020, 18 larvae and 14 adults). Insects were placed individually in Petri dishes layered with two sheets of filter paper. Then, 2000 *Teratorhabditis synpapillata* Sudhaus (Rhabditida: Rhabditidae) CrpA nematodes, which is within the range of nematodes that can be hosted by an adult weevil, suspended in 1 ml of distilled water were added into the Petri plates. Controls received 1 ml of distilled water only. Insects were randomly allocated to the different treatments. The bioassays were maintained at 25℃.

### 2.6 Pathogenicity of *Teratorhabditis synpapillata* on *Galleria mellonella* larvae

To determine the potential of *Teratorhabditis synpapillata* nematodes to affect the lifespan of other insect larvae different than the larvae of the red palm weevils, we evaluated the pathogenicity of *T. synpapillata* against *G. mellonella* larvae. For this, five *G. mellonella* larvae were placed on one Petri dish covered with two sheets of filter paper. Then, 1,000 nematodes suspended in 1 ml of water were added into the Petri plates. Controls received 1 ml of distilled water only. Four independent Petri plates per treatment were assayed (*n* = 4). Insect mortality was recorded daily. Bioassays were maintained at 25℃.

### 2.7 Statistical analysis

Differences in presence of nematodes and fungi in larvae and adults collected in spring 2018 in Ezzahra were analyzed by two‐way ANOVA. The statistical analyses were carried out in Sigma Plot 14.0 (SystatSoftware Inc., San Jose, CA, USA). Normality and equality of variance were verified using Shapiro–Wilk and Levene's tests, respectively. Holm–Sidak post hoc tests were used for multiple comparisons. Insect mortality of red palm weevils collected in Megrine in Autumn 2019, in Ben Arous in Autumn 2020, and of *G. mellonella* larvae were evaluated by log‐rank tests using the “survminer” and “survival” packages in R 4.0.0 and using default parameters in Sigma Plot 14.0 (Kassambara et al., 2017; R Core Team, 2014; Therneau & Lumley, 2015).

## 3 RESULTS

### 3.1 *Rhynchophorus ferrugineus* insects harbor fungi, nematodes, and acari

**FIGURE 2:**
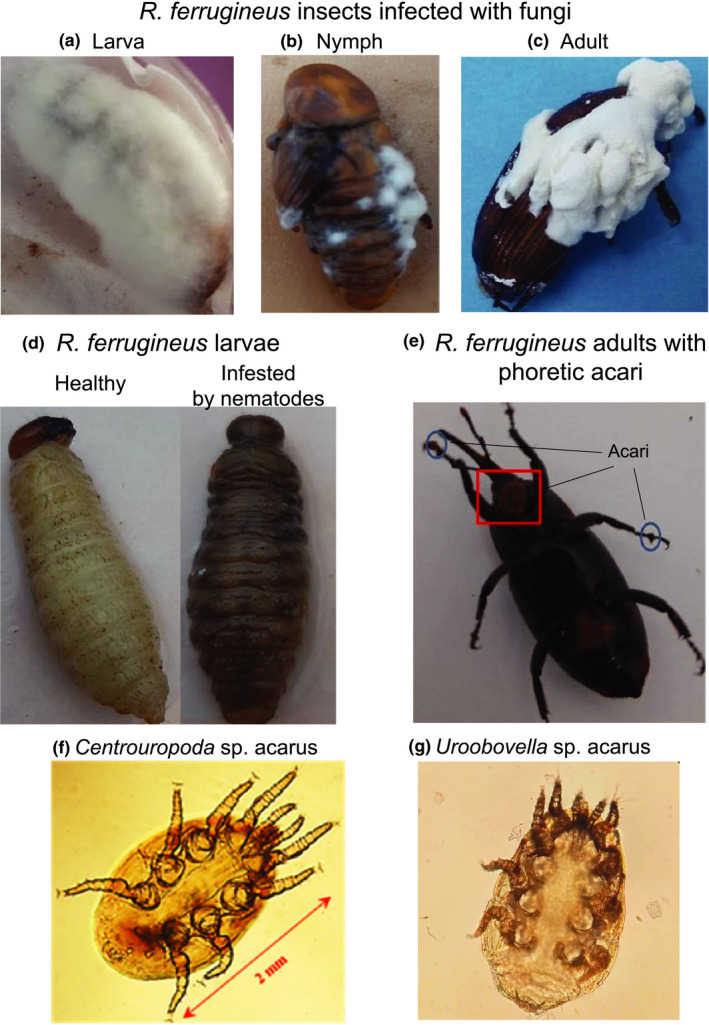
*Rhynchophorus ferrugineus* insects establish close interactions with nematodes, acari, and fungi in natural habitats of Tunisia. (a) larva, (b) nymph, and (c) adult infected with fungi. The larva is fully covered by mycelia. (d) A healthy red palm weevil larva and a larva infested by *Tertorhabditis synpapillata* nematodes. (e) An adult weevil with *Centrouropoda* and *Uroobovella* phoretic acari. (f) *Centrouropoda* sp. acari. (g) *Uroobovella* sp. acari

**FIGURE 3:**
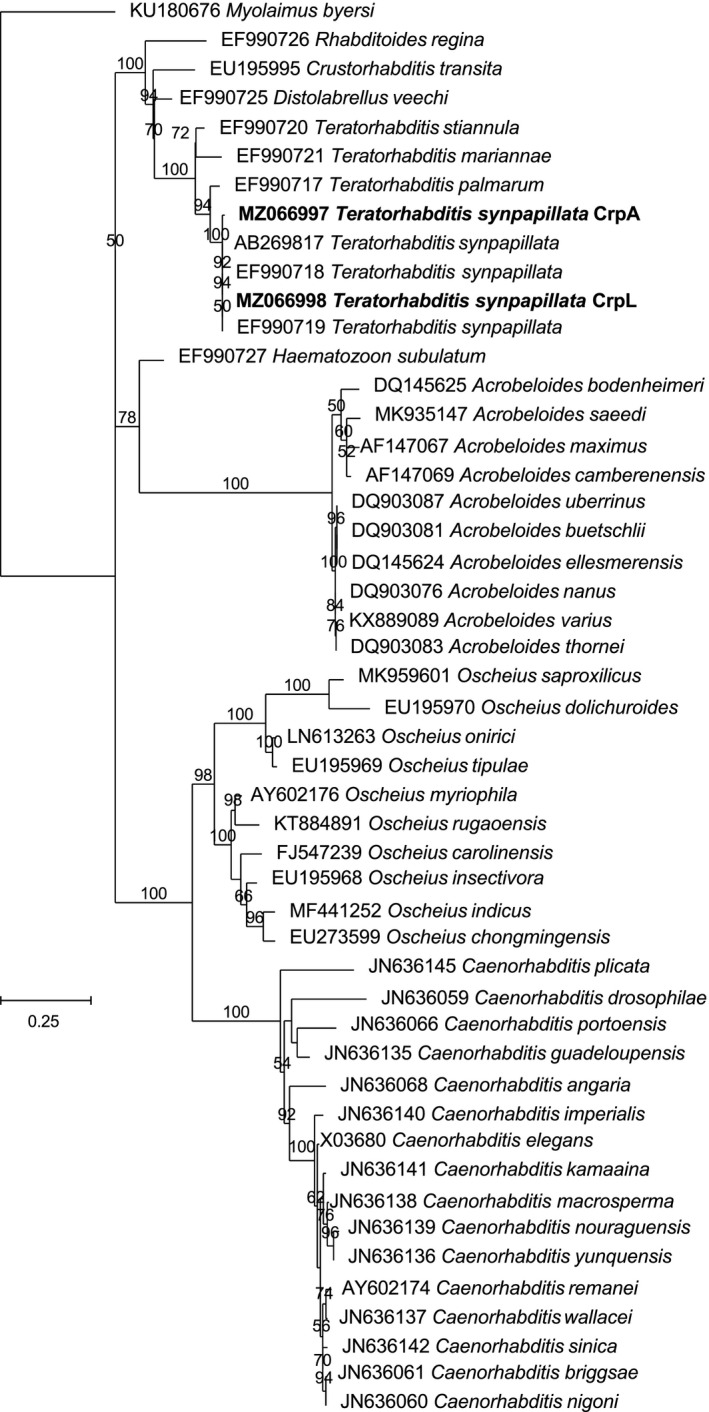
Phylogenetic tree based on ribosomal RNA gene sequences of the nematodes isolated in this study and several related species. Phylogenetic relationships based on the nucleotide sequences of the D2‐D3 expansion segments of the 28S rRNA gene were inferred by using the maximum likelihood method based on the Kimura 2‐parameter model. The tree with the highest log‐likelihood (−6888.80) is shown. The percentage of trees in which the associated taxa clustered together is shown next to the branches. A discrete Gamma distribution was used to model evolutionary rate differences among sites (5 categories (+G, parameter = 0.7836)). The rate variation model allowed for some sites to be evolutionarily invariable ([+I], 15,23% sites). The tree is drawn to scale, with branch lengths measured in the number of substitutions per site. NCBI accession numbers of the sequences used for the analyses are shown

**FIGURE 4:**
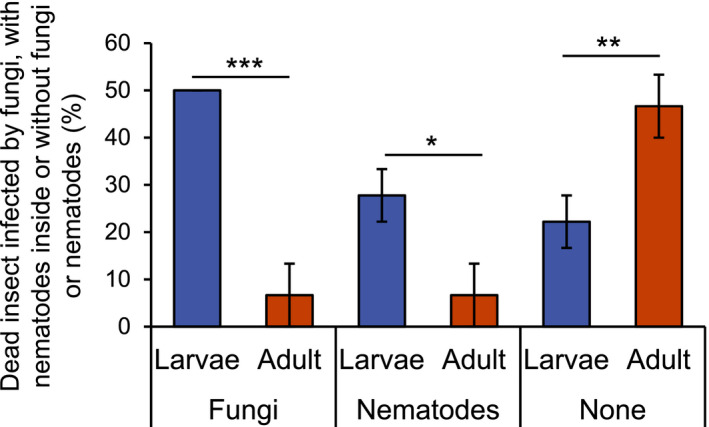
*Rhynchophorus ferrugineus* insects are often infected by fungi or harbor nematodes inside their bodies. Asterisks indicate statistically significant differences (*p < .05, **p < .01, ***p < .001) by two‐way ANOVA. A total of 18 larvae and 15 adults were observed

The red palm weevil insects collected in the cities of Cherguia I and Ben Arous (Figure 1) were carefully inspected and dissected to determine the presence of different organisms that naturally interact with them (Figure 2). We found that some larvae and some adults were infected with fungi and died (Figure 2a–c). In vitro culturing followed by morphological characterization revealed that the fungi correspond to the parasitic fungi *Beauveria bassiana* (Balsamo) Vuillemin (Hypocreales: Cordycipitaceae). We also recovered acari (Figure 2e,f,g). These organisms were found in the thorax, the antennae, and the legs of adults, and none of them were found on the larvae. Morphological characterization revealed that these acari belong to the genus *Centrouropoda* Berlese (Acari: Uropodidae) and *Uroobovella* Berlese (Acari: Uropodidae) (Figure 2f,g). Deeper morphological characterization and/or molecular identification are needed to determine the specific identity of these organisms. Lastly, we also observed that larvae and adults harbor nematodes (Figure 2d, Figure S1). Laboratory colonies of two populations of these nematodes, one isolated from larvae and one isolated from adults, were established. Molecular identification using different regions of the rRNA genes revealed that both belong to the nematode species *Teratorhabditis synpapillata* (Figure 3, Figures S2 and S3). To further characterize these organisms, we observed the larvae and adults collected in spring 2018 in Ezzahra for several days. All insects that died were dissected to determine the presence of nematodes and fungi (Figure 4). Around 50% and 6.5% of the larvae and adults that died, respectively, showed symptoms of infection with the parasitic fungi *Beauvaria bassiana*. Around 28% and 6.5% of the larvae and adults that died, respectively, carried *T. synpapillata* nematodes. Around 22% and 47% of the larvae and adults that died, respectively, did not show any nematodes or fungi (Figure 4). During the observation period, 100% of larvae died, and 60% of adults died.

### 3.2 *Teratorhabditis synpapillata* nematodes reduce lifespan of *Rhynchophorus ferrugineus* larvae but not adults

**FIGURE 5:**
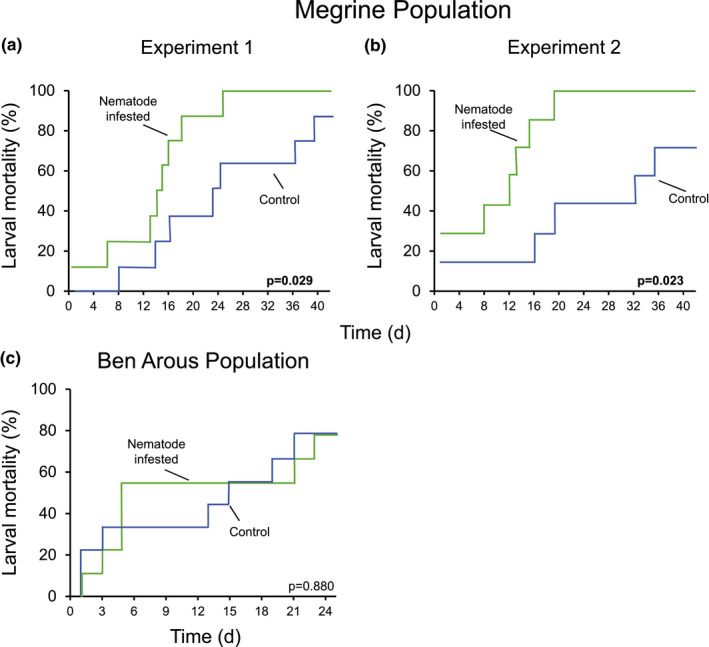
*Teratorhabditis synpapillata* nematodes reduce the lifespan of *Rhynchophorus ferrugineus* larvae. (a) Mortality of control and *T. synpapillata*‐infested larvae collected in Megrine (*n* = 7). (b) Mortality of control and *T. synpapillata*‐infested larvae collected in Megrine (*n* = 8). (c) Mortality of control and *T. synpapillata*‐infested larvae collected in Ben Arous (*n* = 9). Mortality curves were statistically assessed by log‐rank tests

**FIGURE 6:**
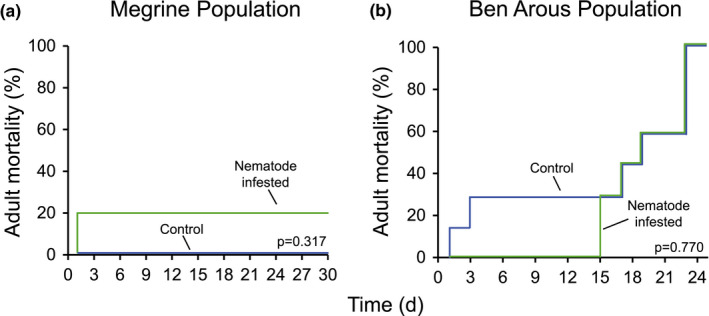
*Teratorhabditis synpapillata* nematodes do not impact the lifespan of *Rhynchophorus ferrugineus* adults. (a) Mortality of control and *T. synpapillata*‐infested adults collected in Megrine (*n* = 5). (b) Mortality of control and *T. synpapillata*‐infested adults collected in Ben Arous (*n* = 7). Mortality curves were statistically assessed by log‐rank tests

To determine whether *T. synpapillata* nematodes can affect the lifespan of red palm weevils and to determine the importance of insect life stage in this context, we infested larvae and adults with *T. synpapillata* nematodes and evaluated mortality over time. Larvae treated with nematodes died faster than control larvae, and this effect was stronger in larvae collected in Megrine than in the larvae collected in Ben Arous (Figure 5, Table 1). In stark contrast, nematodes did not affect lifespan of any of the two populations of red palm weevil adults (Figure 6, Table 1). To determine the specificity of this phenomenon, we infested *G. mellonella* larvae with nematodes. We observed that the lifespan of *G. mellonella* was not affected by the nematodes (Figure S4).

**TABLE 1:** *Teratorhabditis synpapillata* nematodes reduce the lifespan of *Rhynchophorus ferrugineus* larvae, but not of adults

Life stage	Population	Experiment	Treatment	Average lifespan (days)	Reduction in lifespan caused by nematodes
Larvae	Megrine	1	Control	24	37.5%
			Infested	15	
		2	Control	20	35.0%
			Infested	13	
	Ben Arous	1	Control	15	66.6%
			Infested	5	
Adult	Megrine	1	Control	30	0%
			Infested	30	
	Ben Arous	1	Control	19	0%
			Infested	19	

Note

Average lifespan of control and *T. synpapillata*‐infested insects collected at different locations in Tunisia.

## 4 DISCUSSION

*Teratorhabditis synpapillata* nematodes are often isolated from *Rhynchophorus ferrugineus* red palm weevils. They are considered phoretic nematodes as they are normally observed on the bodies of the adults. However, recent studies have also reported the occurrence of these nematodes inside pupae and adults of red palm weevils, suggesting that they can establish other types of relationships apart from phoresy (de Luca et al., 2019; Troccoli et al., 2015). In this study, we found evidence in this context and report that *Teratorhabditis synpapillata* nematodes reduce lifespan of *Rhynchophorus ferrugineus* larvae, but not adults, suggesting then that this nematode–insect relationship is parasitic rather than only phoretic, and uncovers an important factor that regulates population dynamics of this voracious insect pest.

The ecological relevance *R. ferrugineus*–*T. synpapillata* interaction is unclear. On one side, it is often postulated that they establish a phoretic relationship, in which the nematodes can benefit by being dispersed over distances they could hardly cover, and that are transported from the nutrient‐rich soils such as manures to rotten palm trees, that could equally provide them the necessary nutrients for their growth and reproduction (Kanzaki et al., 2008). However, this type of interaction might not provide any benefits for the insects and could even result in detrimental effects, derived for instance from resource competition, as the nematodes and the insects feed from the same food source: rotten palm tissues. In addition, the nematodes might also directly influence life‐history traits of the host once they are transported inside the nest. We tested this hypothesis by observing the lifespan of red palm weevil larvae and adults in direct contact with *T. synpapillata* nematodes. We did not observe any detrimental effect of the nematodes on weevil adults, and however, they reduce the lifespan of larvae by 35%–66%. Our results are consistent with a parasitic rather than a phoretic relationship between these organisms, and uncover a relevant factor that may determine population dynamics of this insects. Might it be that we are evidencing the transition from a free‐living to a parasitic lifestyle of this nematode species? It indeed represents an interesting following up question of our study that deserves further investigation.

## CONFLICT OF INTEREST

None declared.

## AUTHOR CONTRIBUTIONS

**Manel Ibrahim:** Investigation (equal); methodology (equal); writing–review and editing (equal). **Ameni Loulou:** Conceptualization (equal); investigation (equal); methodology (equal); writing–original draft (equal); writing–review and editing (equal). **Anissa Brouk:** Investigation (equal). **Arthur Muller:** Investigation (equal); writing–original draft (equal); writing–review and editing (equal). **Ricardo A. R. Machado:** Conceptualization (equal); formal analysis (equal); funding acquisition (equal); investigation (equal); methodology (equal); project administration (equal); resources (equal); supervision (equal); visualization (equal); writing–original draft (equal); writing–review and editing (equal). **Sadreddine Kallel:** Conceptualization (equal); data curation (equal); formal analysis (equal); funding acquisition (equal); investigation (equal); methodology (equal); project administration (equal); resources (equal); supervision (equal); validation (equal); visualization (equal); writing–original draft (equal); writing–review and editing (equal).

## Supporting information

Supplementary Material

## Data Availability

All data are provided within the manuscript in form of figures/tables. All data are provided as supplementary material (Data S1). Sequences obtained in this study were deposited in the National Center for Bioinformatics for Biotechnology Information (NCBI) databank under accession numbers listed in Table S1.
