# Supplementary material for: Parasites rather than phoronts: Teratorhabditis synpapillata nematodes reduce lifespan of their Rhynchophorus ferrugineus host in a life stage‐dependent manner
Source: Ecol Evol. 2021 Aug 9;11(18):12596–604. doi: 10.1002/ece3.8004 (PMC8462169; doi:10.1002/ece3.8004)
Supplement: Supplementary file 1 — Supplementary Material [file ECE3-11-12596-s001.pdf]

-SUPPLEMENTARY MATERIAL-

Parasites rather than phoronts: Teratorhabditis synpapillata nematodes reduce lifespan of their Rhynchophorus ferrugineus host in a life stage-dependent manner

Manel Ibrahim[1#], Ameni Loulou[1#], Anissa Brouk[1], Arthur Muller[2], Ricardo A. R. Machado[2*], Sadreddine Kallel[1*]

[1] *Université de Carthage, National Agronomic Institute of Tunisia, LR13AGR0, Laboratoire de Recherche Bio-agresseur et Protection Intégrée en Agriculture, 2049 Ariana. Tunisia*

[2] *Experimental Biology Research Group. Institute of Biology. Faculty of Sciences. University of Neuchâtel (UniNE) Rue Emile-Argand 11, 2000 Neuchâtel*

#This author contributed equally to this work.

**Correspondence:* Ricardo A. R. Machado (ricardo.machado@unine.ch) and Sadreddine Kallel (kallel.sadreddine@gmail.com).

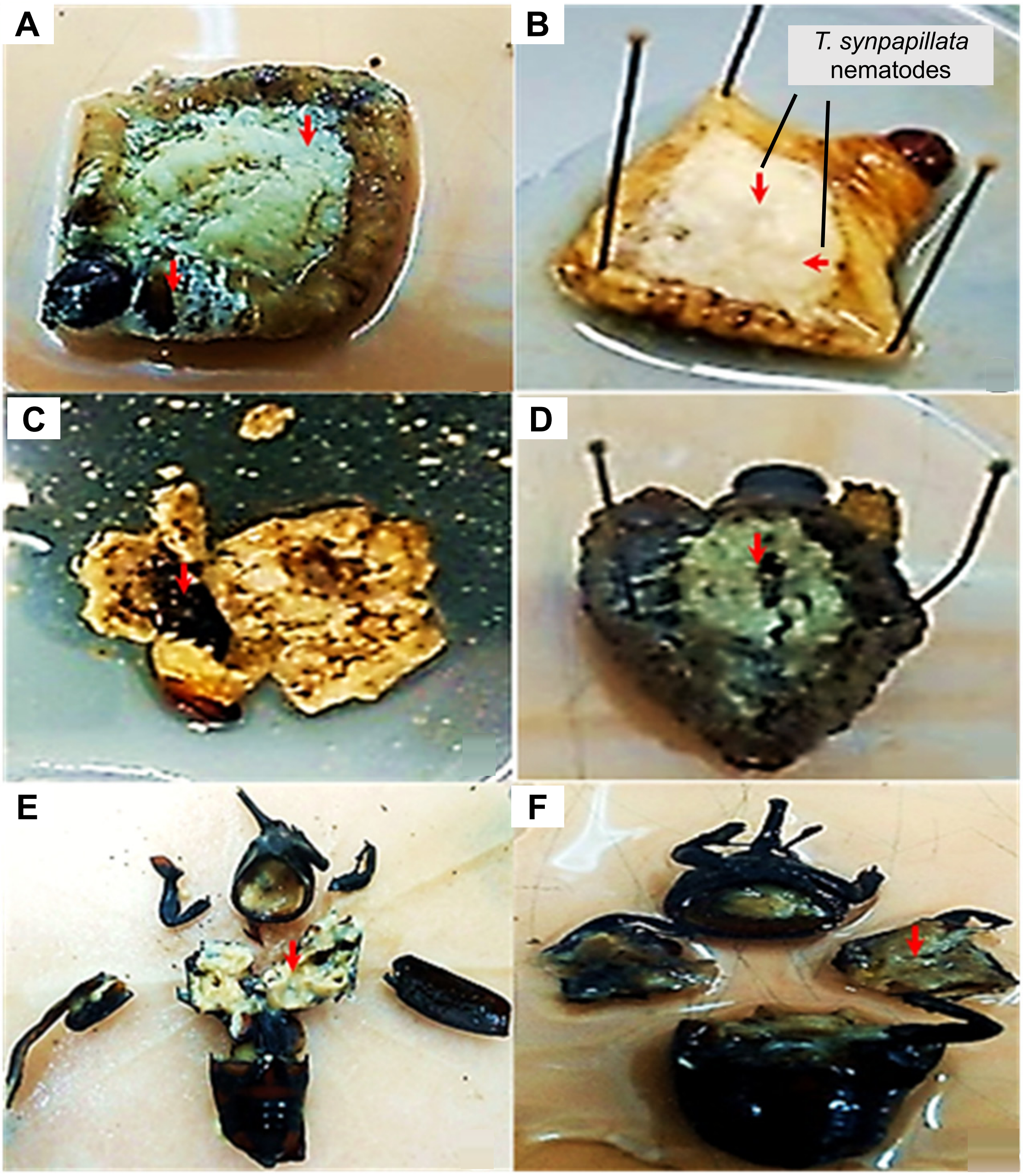

Figure S1 *Wild Rynchophorus ferrugineus adults and larvae harvour Tertorhabditis synpapillata nematodes.* A-D) Dissected larvae showing *T. synpapillata* nematodes, indicated by red arrows, inside their bodies. E-F) Dissected adults showing *T. synpapillata* nematodes, indicated by red arrows, inside their bodies.

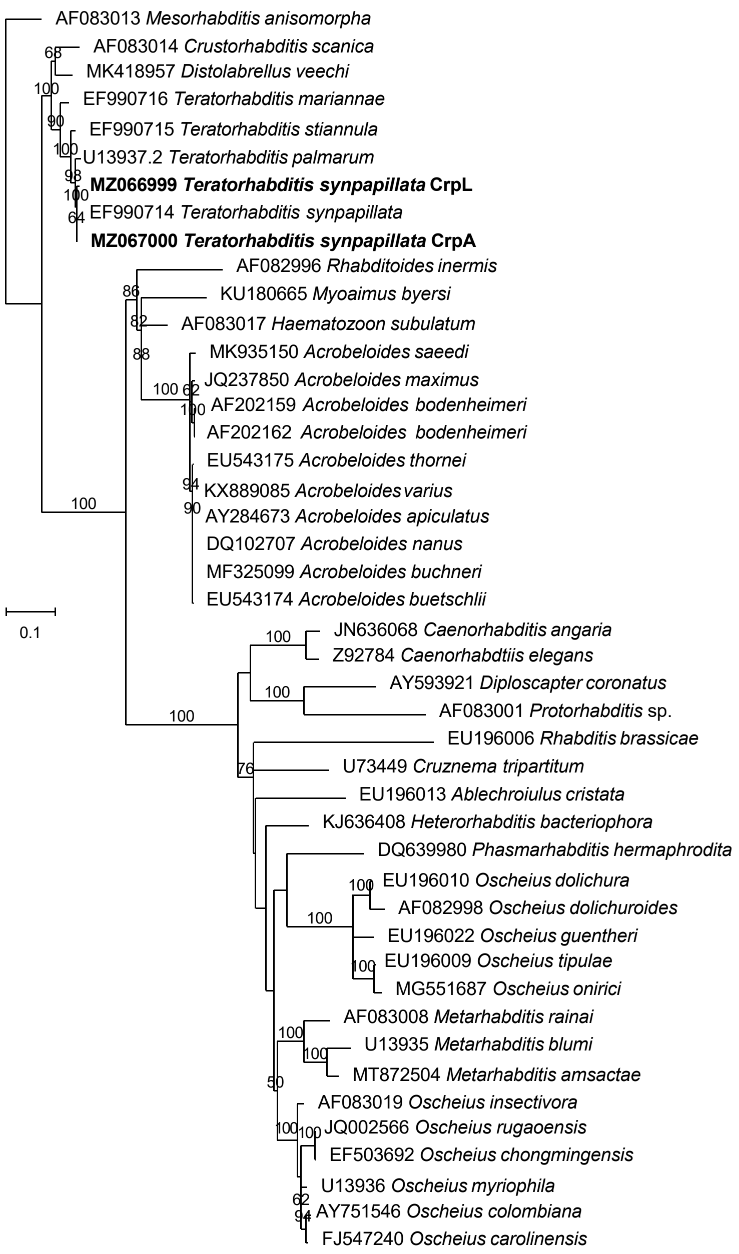

Figure S2 *Phylogenetic tree based on ribosomal DNA sequences of the nematodes isolated in this study and several related species.* Phylogenetic relationships based on 18S rRNA gene sequences were inferred by using the Maximum Likelihood method based on the General Time Reversible model. The tree with the highest log likelihood (-9571.28) is shown. The percentage of trees in which the associated taxa clustered together is shown next to the branches. A discrete Gamma distribution was used to model evolutionary rate differences among sites (5 categories (+*G*, parameter = 0.8439)). The rate variation model allowed for some sites to be evolutionarily invariable ([+I], 17.63% sites). The tree is drawn to scale, with branch lengths measured in the number of substitutions per site. NCBI accession numbers of the sequences used for the analyses are shown.

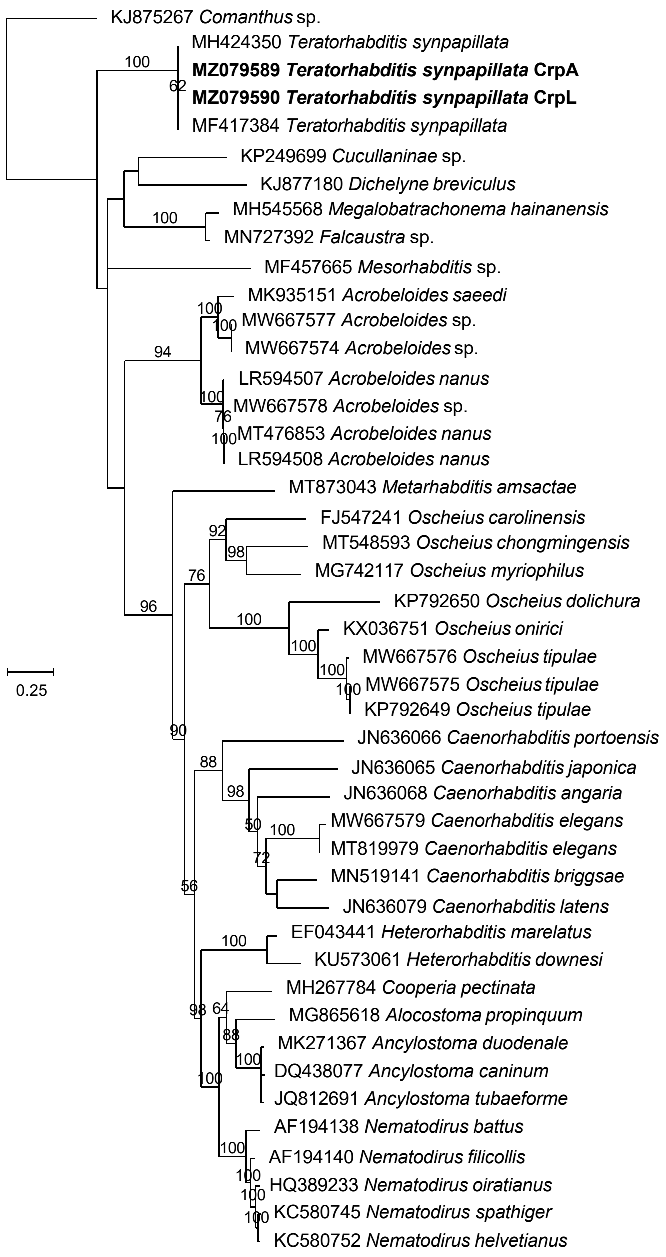

Figure S3 *Phylogenetic tree based on ribosomal RNA gene sequences of the nematodes isolated in this study and several related species.* Phylogenetic relationships based on the nucleotide sequences of the internal transcribed spacer (ITS) region of the rRNA gene were inferred by using the Maximum Likelihood method based on the General Time Reversible model. The tree with the highest log likelihood (-12257.33) is shown. The percentage of trees in which the associated taxa clustered together is shown next to the branches. A discrete Gamma distribution was used to model evolutionary rate differences among sites (5 categories (+G, parameter = 1.9815)). The rate variation model allowed for some sites to be evolutionarily invariable ([+I], 6.71% sites). The tree is drawn to scale, with branch lengths measured in the number of substitutions per site. NCBI accession numbers of the sequences used for the analyses are shown.

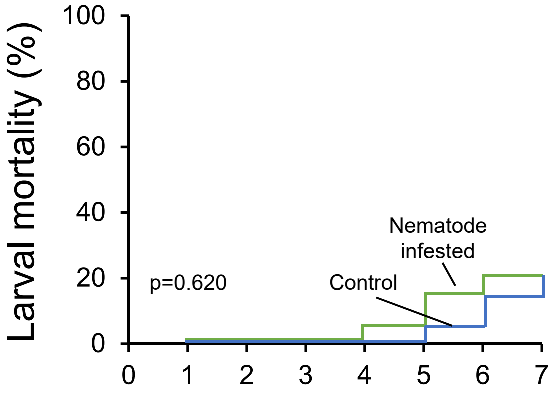

Figure S4 *Teratorhabditis synpapillata nematodes do not influence the lifespan of Galleria mellonella larvae.* Mortality of control and *T. synpapillata*-infested *G. mellonella* (n=4, with five larvae each). Mortality curves were statistically assessed by Log-rank tests.

Supplementary Table 1. NCBI accession numbers of the gene sequences produced in this study.

Organism	rRNA region	NCBI accession number
Teratorhabditis synpapillata CrpA	18S	MZ067000
Teratorhabditis synpapillata CrpL	18S	MZ066999
Teratorhabditis synpapillata CrpA	D2D3	MZ066997
Teratorhabditis synpapillata CrpL	D2D3	MZ066998
Teratorhabditis synpapillata CrpA	ITS	MZ079589
Teratorhabditis synpapillata CrpL	ITS	MZ079590
